# The Australia-modified Karnofsky Performance Status (AKPS) scale: a revised scale for contemporary palliative care clinical practice [ISRCTN81117481]

**DOI:** 10.1186/1472-684X-4-7

**Published:** 2005-11-12

**Authors:** Amy P Abernethy, Tania Shelby-James, Belinda S Fazekas, David Woods, David C Currow

**Affiliations:** 1 Department of Palliative and Supportive Services, Division of Medicine, Flinders University, Bedford Park, South Australia, Australia; 2 Southern Adelaide Palliative Services, Repatriation General Hospital, Daw Park, South Australia, Australia; 3 Division of Medical Oncology, Department of Medicine, Duke University Medical Center, Durham, North Carolina, USA; 4 North Tasmanian Palliative Care Service, Launceston, Tasmania, Australia

## Abstract

**Background:**

The Karnofsky Performance Status (KPS) is a gold standard scale. The Thorne-modified KPS (TKPS) focuses on community-based care and has been shown to be more relevant to palliative care settings than the original KPS. The Australia-modified KPS (AKPS) blends KPS and TKPS to accommodate any setting of care.

**Methods:**

Performance status was measured using all three scales for palliative care patients enrolled in a randomized controlled trial in South Australia. Care occurred in a range of settings. Survival was defined from enrollment to death.

**Results:**

Ratings were collected at 1600 timepoints for 306 participants. The median score on all scales was 60. KPS and AKPS agreed in 87% of ratings; 79% of disagreements occurred within 1 level on the 11-level scales. KPS and TKPS agreed in 76% of ratings; 85% of disagreements occurred within one level. AKPS and TKPS agreed in 85% of ratings; 87% of disagreements were within one level. Strongest agreement occurred at the highest levels (70–90), with greatest disagreement at lower levels (≤40). Kappa coefficients for agreement were KPS-TKPS 0.71, KPS-AKPS 0.84, and AKPS-TKPS 0.82 (all p < 0.001). Spearman correlations with survival were KPS 0.26, TKPS 0.27 and AKPS 0.26 (all p < 0.001). AKPS was most predictive of survival at the lower range of the scale. All had longitudinal test-retest validity. Face validity was greatest for the AKPS.

**Conclusion:**

The AKPS is a useful modification of the KPS that is more appropriate for clinical settings that include multiple venues of care such as palliative care.

## Background

Palliative care clinicians are increasingly using change in performance status as a flag for likelihood of need for services, timing of interventions, and as an outcome measurement for clinical programs and research [1-3]. The Karnofsky Performance Scale (KPS) has been used as an assessment tool for performance status in oncology since 1948 [4]. It is commonly regarded as the gold standard measurement of performance status in cancer[2,3]. The KPS scale assesses three dimensions of health status – activity, work and self-care – and can be administered by any healthcare professional for a quick assessment of general functioning and survival [5].

The original KPS is an ordered categorical scale with 11 levels (Table 1). Extensive psychometric testing provides evidence of acceptable reliability and validity in patients with cancer[3,6,7]. The KPS correlates well with physical functioning, such as walking and stair climbing [7]. It has been repeatedly demonstrated to be useful in assisting prognostication [4,8-11]. In an evaluation of predictive validity, Mor *et al *found significant correlation between KPS at initial interview and survival time (r = 0.30, P < 0.001) [6]. When KPS is low it is a sensitive predictor of poor prognosis, but when high it is a poor cross-sectional indicator of prognosis [2].

**Table 1:** Comparison of the original Karnofsky Performance Status Scale (KPS), Thorne-modified Karnofsky Performance Status Scale (TKPS), and the Australia-modified Karnofsky Performance Status Scale (AKPS). Italicised areas reflect the original KPS instrument.

**Score (Category)**	***Original Karnofsky (KPS)***	**Thorne-modified Karnofsky (TKPS)**	**Australia-modified Karnofsky (AKPS)**
100 (A)	*Normal; no complaints; no evidence of disease*.	*Normal; no complaints; no evidence of disease*.	*Normal; no complaints; no evidence of disease*.
90 (A)	*Able to carry on normal activity; minor signs or symptoms*.	*Able to carry on normal activity; minor signs or symptoms*.	*Able to carry on normal activity; minor signs or symptoms*.
80 (A)	*Normal activity with effort; some signs or symptoms of disease*.	*Normal activity with effort; some signs or symptoms of disease*.	*Normal activity with effort; some signs or symptoms of disease*.

70 (B)	*Cares for self; unable to carry on normal activity or to do active work*.	*Cares for self; unable to carry on normal activity or to do active work*.	*Cares for self; unable to carry on normal activity or to do active work*.
60 (B)	*Requires occasional assistance but is able to care for most of his needs*.	Requires professional visits less than once a week.	*Requires occasional assistance but is able to care for most of his needs*.
50 (B)	*Requires considerable assistance and frequent medical care*	Requires professional visits more than once a week.	*Requires considerable assistance and frequent medical care*

40 (C)	*Disabled; requires special care and assistance*.	In bed more than 50% of the time.	In bed more than 50% of the time.
30 (C)	*Severely disabled; hospitalisation necessary; active supportive treatment is necessary*.	Almost completely bedfast.	Almost completely bedfast.
20 (C)	*Very sick; hospitalisation necessary; active supportive treatment is necessary*.	Totally bedfast and requiring extensive nursing care by professionals and/or family.	Totally bedfast and requiring extensive nursing care by professionals and/or family.
10 (C)	*Moribund; fatal processes progressing rapidly*.	Comatose or barely arousable.	Comatose or barely arousable.

0	*Dead*.	*Dead*.	*Dead*.

While useful, the original KPS has limitations. It was based on the models of health service delivery available in 1948, linking performance status with strict recommendations about where further clinical care should be provided (Table 1). At KPS 30 and below there are recommendations for the intensity of clinical care including hospitalization. In particular, the KPS focus on need for hospitalization and medical intervention limits its applicability in care settings where clinical options extend to non-hospital-based care directed at support rather than cure, such as palliative care settings. The language may be uncomfortable or confusing to nurses and other clinicians expected to apply the scale to palliative patients for whom they are caring in home or hospice settings but then expected to blatantly ignore the KPS recommendations of hospitalization. Recommendations about place of care need not be part of the KPS for it to be clinically useful in the 21^st ^century [3].

These limitations have prompted modifications of the scale and development of new tools that better reflect clinical functioning and variations in place of care for palliative care patients. In the late 1990's, Thorne developed a modified version of the KPS (Thorne-modified Karnofsky Performance Status, TKPS, Table 1) for use in palliative home care settings. The TKPS reworded the categories at the lower end of the KPS scale to correlate with professional care needs and activity, removing references to location of care. The TKPS was validated by Nikoletti and colleagues in a sample of 78 Australian home-hospice patients [3].

While the TKPS was found to be more applicable for home-based hospice settings, it was limited in its use for hospitalized palliative care patients. In response, the original KPS and the newer TKPS were melded into a single scale that accommodated all of the venues of clinical palliative care – the Australia-modified Karnofsky Performance Status Scale (AKPS, Table 1). In the AKPS, the TKPS link to health professionals' visits was avoided, favoring a generic approach focusing on function alone.

We conducted a large randomized controlled trial that incorporated performance status as a main outcome measure – the Palliative Care Trial. This study is a 2 × 2 × 2 factorial cluster randomized controlled trial involving 461 consenting patients and their general practitioners (GPs) recruited from April 2002 and June 2004. Participants were randomized to case conferences, GP pain education, and patient pain education. Patient participants were cared for in a variety of clinical settings consistent with contemporary clinical practice. It was important that the performance status scale used was appropriate and carefully validated. The full clinical trial methodology has been presented elsewhere [1].

The purpose of this current study was to determine the performance status measure most appropriate for the clinical and research needs of the community based palliative care service conducting the Palliative Care Trial. The expected goals were to do the following:

1. To determine if the TKPS and AKPS had similar predictive values for survival as compared with the KPS in a contemporary specialized palliative care service that incorporates a range of clinical settings including acute hospital care, inpatient hospice, community care, and aged care facilities;

2. To determine if one version of the KPS instrument was clearly superior to the others in this setting;

3. If equal, to determine which instrument was most acceptable to clinical and research staff and easiest to use; and,

4. To document reliability of the three instruments in the local clinical setting.

## Methods

This current study was an embedded sub-study within the Palliative Care Trial [1]. It was decided *a priori *that after KPS, TKPS and AKPS data were collected from at least 120 participants who exited the trial, a validity assessment of the scales would be conducted. Based on this analysis all subsequent Palliative Care Trial assessments would include only the most reliable of these 3 measures.

### Study setting

The trial was set in Adelaide, South Australia and based at Southern Adelaide Palliative Services. GPs are the primary point of care for palliative patients. Specialized palliative care services funded by the state government provide consultative specialist medical and nursing support for GPs and community nurses. Referrals to the palliative care service come from health professionals, family and patients, and nearly all palliative care patients within the region are referred to the same geographic service. The indication for, and timing of referral varies; indications include physical symptom control, and the need for coordinated and multi-disciplinary care. Patients frequently receive active therapy such as radiotherapy and chemotherapy in parallel with palliative care services. The model of care is consistent with the definition of palliative care described in the 2004 United States (US) National Consensus Project Clinical Practice Guidelines for Quality Palliative Care [12].

Southern Adelaide Palliative Services is a comprehensive program with medical specialists, nursing specialists, social work, inpatient care, community and outpatient visits, home care, nursing home consultations, a bereavement program, volunteers, and complementary care. Venues of care include community, acute inpatient, sub-acute inpatient, inpatient hospice, respite, nursing home, and hostel settings. There were 1,094 new referrals in 2003, 85% of whom had cancer. The mean time from referral to death was 119 days with a median of 47 days. Ten percent of referrals were from GPs, 50% from local hospitals, 20% from medical specialists, and 10% from district nurses. Ninety percent of patients spent some time in the hospital in the last year of life and less than one third spent any time in the inpatient hospice.

### Study participants

All adult patients referred to Southern Adelaide Palliative Services with any form of pain in the preceding three months were eligible for the Palliative Care Trial. This definition was very broadly applied and could refer to any type of pain. This pain could have been temporary and not related to the predominate illness, but would provide a reference point for pain assessments during the research study. Patients who did not live within the geographic region served by the palliative care service were excluded. Patients who were expected to die within 48 hours of referral were also excluded, since the recruitment process of consenting both the patient and GP was expected to take two days. Participants must have been mentally competent at enrollment as documented by a Folstein Mini-mental Status Examination (MMSE) Score = 24 [13], or have a GP-identified caregiver or legal healthcare proxy who could adequately provide informed consent [14]. Patients unwilling to provide their contact information to the trial staff in order to learn more about the study were not enrolled. All GPs and GP practices of consenting eligible patients were eligible. Both patient and GP consent were required for enrollment and randomization.

### Measures

Patient participant functional status was assessed by the KPS, TKPS and AKPS (Table 1) simultaneously. For those patients who died during the trial, survival was defined as time from consent to participate in the trial until death.

### Study procedures

KPS, TKPS and AKPS were collected at every clinical encounter and data collection time point (minimally baseline and every 2 weeks for the first 12 weeks then monthly until death or exit from the trial) for the first 300 participants randomized in the trial. Based on our sample size assumptions and recruitment estimates, this would provide data for over 120 participants who exited the trial through either death or withdrawal from the study. Palliative care clinical nurses collected all of the data within the study. Data collection was incorporated into regular palliative clinical visits irrespective of location of care (hospital- or community-based care). In total, 26 full or part-time nurses collected data.

All clinical nurses participated in training in performance status assessment. This included at least a 30-minute review of the scales, data collection forms, and reasons for using all three measures. In addition, written instructions were prepared that included a list of example questions that could be used to determine performance status classification. Nurses were provided laminated cards with each of the scales. Responses were captured on a standardized set of study forms. Nurses also underwent more specific training on data collection methods, the importance of data quality, communication and research ethics as part of the Palliative Care Trial [1].

### Analysis

Descriptive statistics were used to summarize the participant population and performance status scores observed. Agreement between the KPS, TKPS and AKPS was assessed by a variety of methods. Percentage agreements and disagreements were calculated; overall agreement and proportion of disagreements at one, two or three levels on the scales were reported in the method of Nikoletti *et al *[3]. Kappa statistics were calculated in order to exclude agreement due to chance. Simple kappa statistics were reported and subjected to the following interpretation: 0.81 to 1.00 almost perfect agreement, 0.61 to 0.80 substantive agreement, 0.41–0.60 moderate agreement, 0.21–0.40 fair agreement, 0.00–0.20 slight agreement, and less than 0.00 poor or no agreement [15]. Bland-Altman plots of the difference between paired scores versus the mean of the scores were used to assess agreement [16]. The association between performance status and survival was determined using Spearman correlation coefficients [5]. Actuarial survival was estimated using Kaplan-Meier methods and reported as median (interquartile range (IQR)) survival [17]. Between group comparisons were made using the log rank test [18]. All analyses were conducted with the SAS System (Version 9.1, Cary, North Carolina, USA).

### Ethics approval and trial registration

The Palliative Care Trial was approved by all twelve relevant independent Institutional Review Boards (IRBs) and Human Research and Ethics Committees (HRECs) including the Australian Department of Veterans Affairs and Health Insurance Commission, Canberra, Australia. This sub-study was included in the approved protocols and participant consent forms. The Palliative Care Trial is registered with the ISRCTN – ISRCTN81117481 .

## Results

KPS, TKPS and AKPS were assessed simultaneously 1600 times in a total of 306 patients. There were a mean of 5.3 (standard deviation (SD) 5.0) assessments per individual, with median 4.0 (range 1–44). Within the same individual, the multiple performance assessments were collected over a mean of 76 (SD 88) days, with median 49 (range 0–507). Seventy-eight percent of assessments were done in the patient's home, 7% in an aged care facility, 5% in another relative's home, 5% in the hospital, 4% in the inpatient hospice unit, and 1% in another location of care.

The baseline characteristics of the patient population are presented in Table 2. The mean age of the study population was 71 years, 148 (49%) were male, 190 (63%) married, and 69 (23%) widowed. The majority (93%) had cancer and had a primary caregiver (96%). Fourteen patients had a MMSE score of <24; of those, 10 had an AKPS of <60.

**Table 2:** Baseline participant characteristics

**Characteristic**	**Category**	**N**	**%**
Gender	Male	148	49%
			
Age (Mean/SD)		71	12%
			
Marital status	Never Married	9	3%
	Widowed	69	23%
	Divorced/Separated	33	11%
	Married/Defacto	190	63%
			
Caregiver present	Has caregiver	277	96%
			
Accommodation	Private residence	271	89%
	Nursing home	18	6%
	Hostel	4	1%
	Hospital	11	4%
	Other	2	1%
			
Living arrangement	Lives alone	72	25%
	Lives with spouse	173	60%
	Other relative lives in household	44	15%
			
Cancer diagnosis	Yes	282	92%
			
Phase of palliative care	Stable	157	60%
	Deteriorating	48	18%
	Unstable	57	22%
	Terminal	1	0%
			
Pain at present	Mean (standard deviation)		(2.1)
Usual pain in last 24 hrs			(2.1)
Worst pain in last 24 hrs			(3.2)

**Figure 1:**
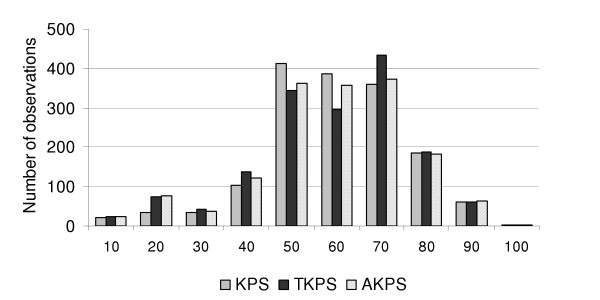
Profile of KPS, TKPS and AKPS scores in the 1600 observations.

The profile of KPS, TKPS and AKPS scores in is shown in Figure 1. The majority of scores centered around 50–70 (group B). Measures of central of central tendency are presented in Table 3; the median score was 60 on all three scales.

**Table 3:** Measures of central tendency and dispersion for the KPS, TKPS and AKPS scores

	**N**	**Mean**	**Median**	**Mode**	**SD**	**Min-Max**
**KPS**	1600	59.7	60	50	15.7	10–100
**TKPS**	1600	59.0	60	70	17.3	10–100
**AKPS**	1600	58.7	60	70	17.1	10–100

**Figure 2:**
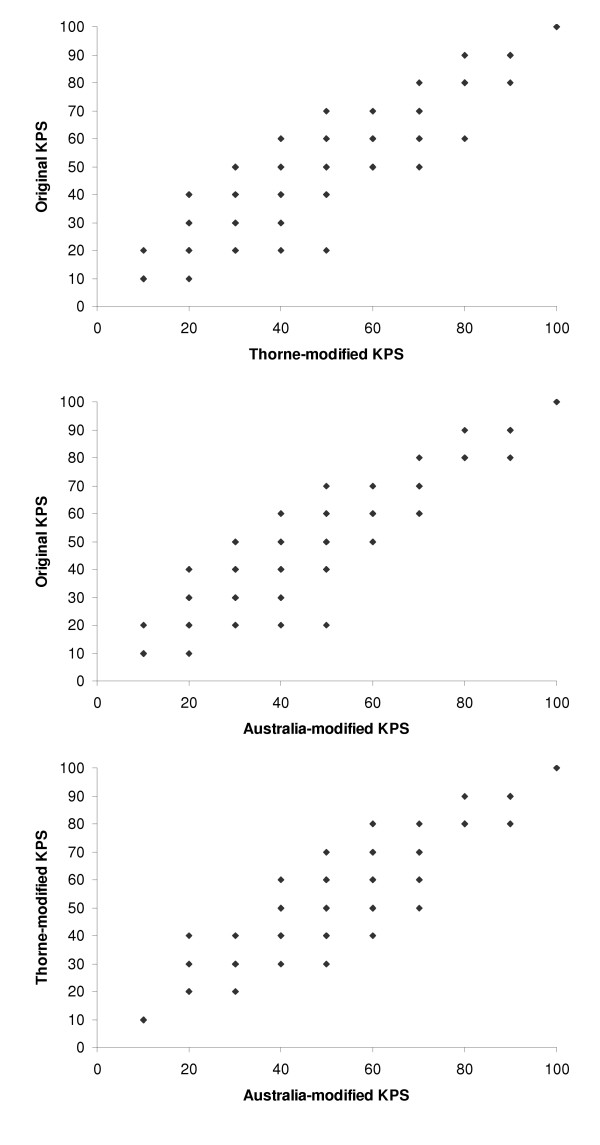
Scatter plots of KPS-TKPS, KPS-AKPS, and AKPS-TKPS pairs.

**Figure 3:**
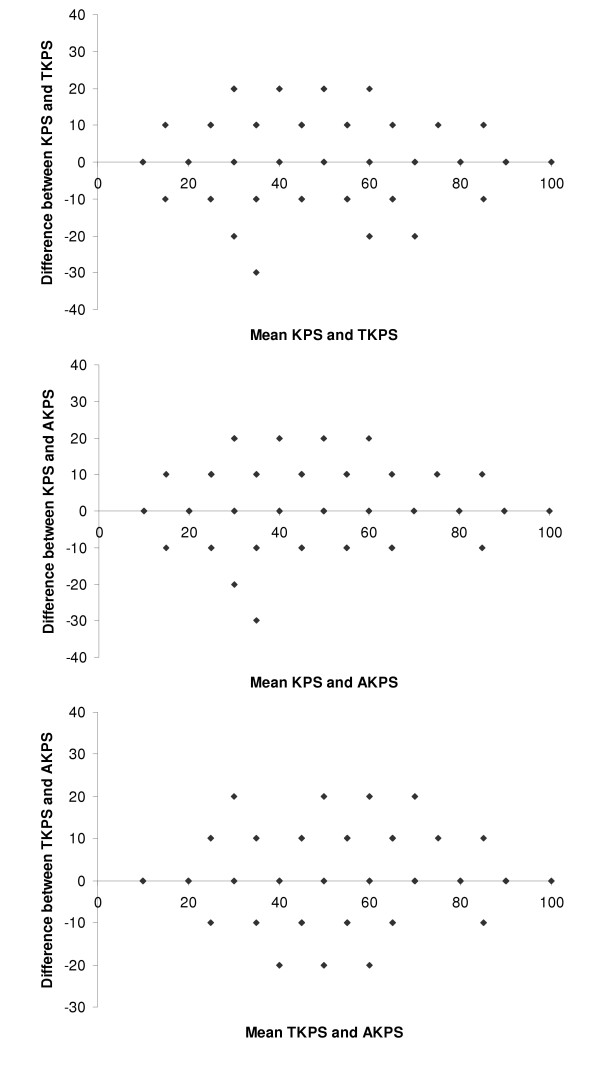
Bland & Altman plot for the performance status pairs.

The agreement between scores was calculated for KPS-TKPS, KPS-AKPS and TKPS-AKPS pairings, as shown in Table 4; relevant scatter plots are in Figure 2. These demonstrate a high level of agreement between the 3 scales. When disagreement existed, KPS scores were more commonly higher than the TKPS or AKPS scores; the profile of disagreement between TKPS and AKPS was more evenly split and nearly always confined to one level. Bland-Altman plot presented in figure 3 verify these interpretations.

**Table 4:** Comparison between KPS, TKPS and AKPS scores by levels

	**N**	**%**
**Comparison between scores for KPS and TKPS**		
*By score*		
TKPS > KPS by three levels	1	0.1%
TKPS > KPS by two levels	7	0.4%
TKPS > KPS by one levels	149	9.3%
Complete agreement	1216	76.0%
TKPS < KPS by one levels	179	11.2%
TKPS < KPS by two levels	48	3.0%
TKPS < KPS by three levels	0	0.0%
*By Group (ABC)*		
TKPS >KPS by one group	96	6.0%
Complete agreement	1489	93.1%
TKPS <KPS by one group	15	0.9%

**Comparison between scores for KPS and AKPS**		
*By score*		
AKPS > KPS by three levels	1	0.1%
AKPS > KPS by two levels	1	0.1%
AKPS > KPS by one levels	44	2.8%
Complete agreement	1386	86.6%
AKPS < KPS by one levels	123	7.7%
AKPS < KPS by two levels	45	2.8%
AKPS < KPS by three levels	0	0.0%
*By Group (ABC)*		
AKPS >KPS by one group	80	5.0%
Complete agreement	1507	94.2%
AKPS <KPS by one group	13	0.8%

**Comparison between scores for TKPS and AKPS**		
*By score*		
TKPS > AKPS by three levels	0	0.0%
TKPS > AKPS by two levels	8	0.5%
TKPS > AKPS by one levels	139	8.7%
Complete agreement	1359	84.9%
TKPS < AKPS by one levels	88	5.5%
TKPS < AKPS by two levels	6	0.4%
TKPS < AKPS by three levels	0	0.0%
*By Group (ABC)*		
TKPS >AKPS by one group	15	0.9%
Complete agreement	1556	97.3%
TKPS <AKPS by one group	29	1.8%

Disagreements tended to cluster at the mid-point and lower potions of the scales as shown in the scatter plots of Figure 2. The Kappa coefficient for agreement between all KPS and TKPS measurements was 0.71 (p < 0.001), between all KPS and AKPS measurements was 0.84 (p < 0.001), and between all AKPS and TKPS measurements was 0.82 (p < 0.001).

**Figure 4:**
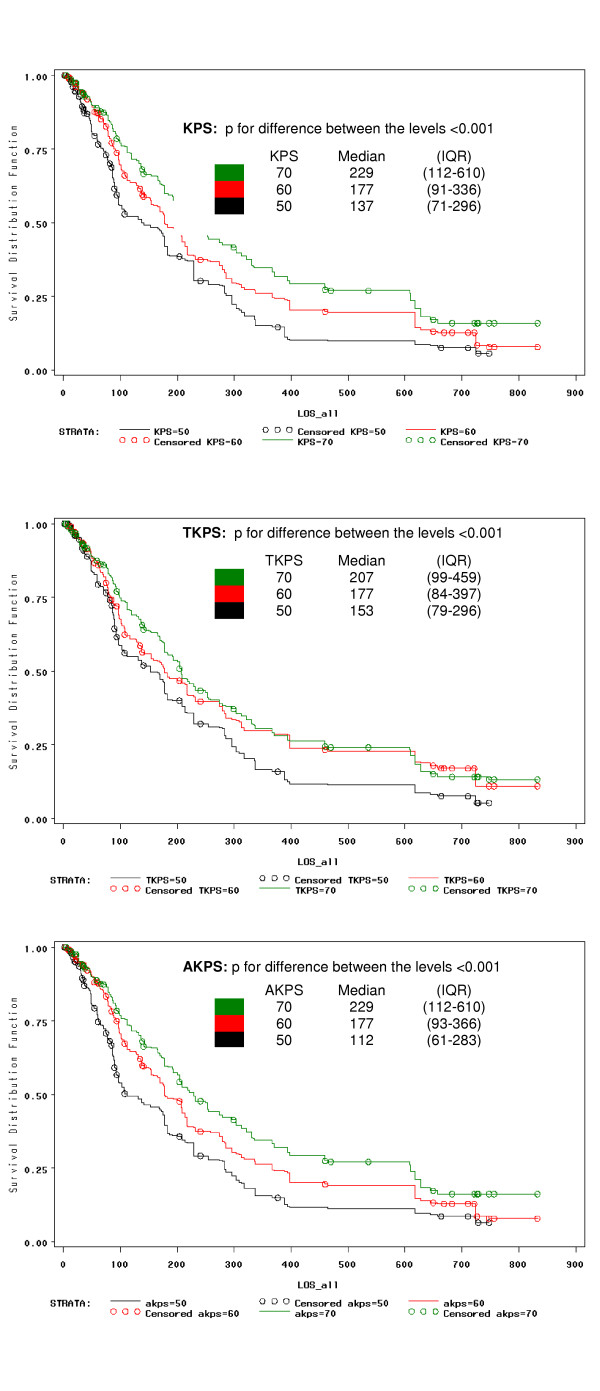
Survival probabilities according to KPS, TKPS and KPS by levels 50, 60 and 70 (Category B).

**Figure 5:**
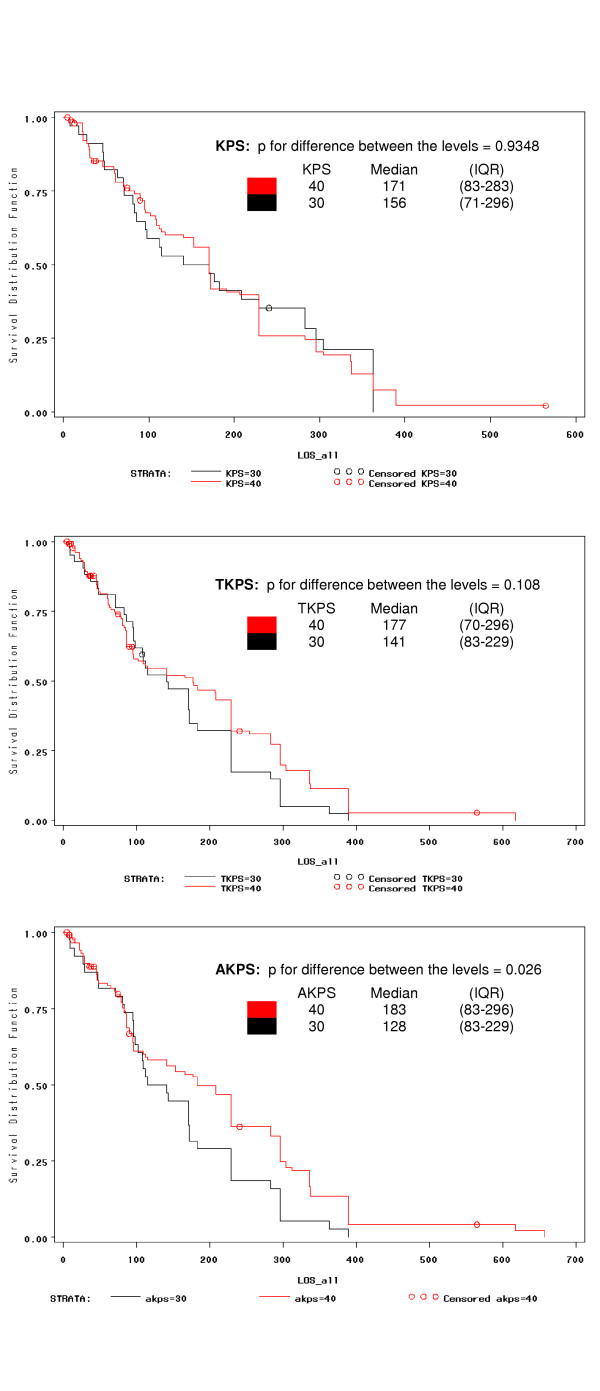
Survival probabilities according to KPS, TKPS and KPS by levels 30 and 40 (Category C).

All three performance measurements were correlated with survival. For those participants who died while on the trial (n = 232), the Spearman correlation coefficients of baseline KPS, TKPS, and AKPS and overall survival were 0.26, 0.27, and 0.26 respectively all with p < 0.001. These positive correlations imply that when performance status increased, survival also increased. Since palliative patients are more likely to have poor performance status, the predictive power of the three instruments in the intermediate (category B) and lower (category C) ranges was investigated (Figures 4 and 5). For category B, all three instruments clearly discriminated survival for levels 50, 60 and 70 (p < 0.001; Figure 4). For category C, only AKPS could significantly discriminate survival based upon AKPS levels of 30 and 40 (p = 0.026; Figure 5).

**Figure 6:**
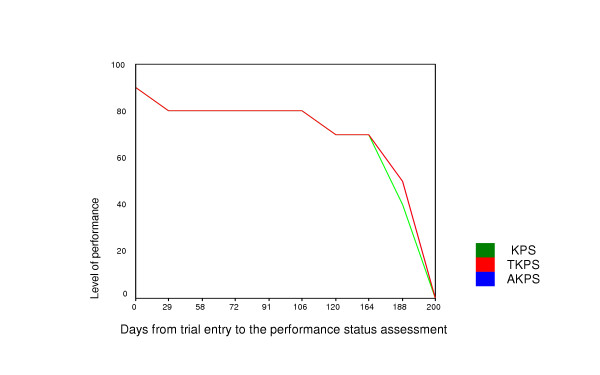
Demonstration of the ability of the three instruments to respond to change in performance status over time. Plots nearly overly each other. Similar plots were generated for all participants.

Longitudinal test-retest reliability indicates the ability to have multiple longitudinal assessments that are sensitive to meaningful change in the outcome. Since we would be measuring performance status over time in the trial, this was of considerable concern for us. KPS, TKPS, and AKPS longitudinal curves were generated for all participants; an example is given in Figure 6. Longitudinal trends consistent with the established palliative care trajectories of illness were observed with all instruments (data not shown). Change in performance status level was investigated for each participant when moving from the stable to the deteriorating phase of palliative care (n = 96). Palliative care phase is predictive of the need for palliative care service intervention and resource utilization [19,20]. When phase deteriorated, KPS, TKPS and AKPS all decreased by a median of 10 (range -10 to 50).

Face validity was assessed by asking nurses to indicate which scale was easiest to use and best suited the patient population. Nurses involved in the collection of data for the trial were highly skilled and experienced in palliative care with a mean length of time in nursing of 18 years (range 6–29) with an average of 9 years (range 2–16) in palliative care. All nurses had university qualifications; 24 of the nurses were employed at RN level 2 (consultant) and 2 employed at RN Level 3 (consultant and managerial). All 26 nurses preferred using the AKPS as they felt that the categories were more consistent with the multiple venues of care and different levels of interventions needed in their current clinical practice.

## Discussion

KPS is a widely used measure demonstrated to have important correlations with both resource utilization and prognosis at the end of life. Generating a measure of function with language that is consistent with current clinical practice is crucial for the ongoing use of KPS. For the measure to transcend differences in funding of healthcare and models of service delivery, it is timely to remove specific references to the place or intensity of care and focus on function alone.

The Thorne modification developed in the 1990's was an important validated update, making the scale useful for contemporary palliative home care settings, especially hospice. The TKPS concentrated on the community setting, though, limiting the scale's utility in the varied clinical settings encountered in palliative care including inpatient hospice, acute inpatient care, and nursing home care. This is the first research report of the Australia-modified version (AKPS), an important amalgam of the original KPS and the TKPS applicable to both inpatient and community palliative care. The categories in the AKPS are less directive of the expected location of care; however, as much of the original KPS and TKPS language as possible has been maintained in order to reduce confusion and the need for extensive retraining for clinicians already familiar with the earlier versions.

In this study all versions of the KPS could be used in the various venues of palliative care including the community, acute inpatient, subacute inpatient, inpatient hospice, respite, nursing home, and hostel settings. AKPS had the highest agreement with both KPS and TKS (Table 4, Figures 2 and 3), and was equally predictive of survival (Figure 4). When considering the lower end of the scale (category C) where more palliative patients cluster, AKPS was most predictive of survival (Figure 5). All scales were able to reflect longitudinal change. The nurses reflected that AKPS was easiest to use and most acceptable. This study demonstrated that AKPS had excellent correlation with the original KPS while allowing for palliative care sensitive clinician responses to changes in level of function as death approaches, both in terms of place of care and the clinical staff who need to be involved in that care. The better performance of the AKPS will assist with better decision-making in palliative care.

The high level of agreement among the three versions was expected, given the similarity of the three scales. However, before a new scale is adopted for day-to-day clinical practice it is important that it is carefully and prospectively evaluated to ensure that the results reflect what the user expects to be measuring. Further, as we planned to use performance status as a primary outcome in a major clinical trial in palliative care it was vital to verify the validity of the AKPS as an outcome measure within the palliative care setting before limiting all of our data collection to this single measure. The need for formal validation is evident in Table 4 and Figure 2. Some participants were assessed as a KPS of 20 and an AKPS of 50 even when the KPS 50 and AKPS 50 had exactly the same phrasing. This was done by the same nurse assessing the patient using each of the scales sequentially at the same evaluation visit. This difference in scoring was reflective of the difference in phrasing at other levels on the scales. For an individual palliative care patient, a score of KPS 20 ("very sick; hospitalization necessary; active supportive treatment necessary") may be the best option on that scale, however when reviewing the AKPS scale the score of 50 ("requires considerable assistance and frequent medical care") was more appropriate in relation to all other levels on the scale. Importantly, the AKPS instrument with more palliative care appropriate language did not alter the scale's expected overall correlation with survival, was more correlated with survival at lower performance status, and was more acceptable to the clinical nurses.

### Longitudinal assessment

Any of the KPS tools provide both an objective measure of the current status of the person being assessed and useful trends when used longitudinally. Longitudinal trends in performance status is an important aspect of prognostication for the longer term outlook of the patient including his or her anticipated health resource and service needs over time, as demonstrated by the relationship between performance status and phase of palliative care [20]. Such changes in level of function reflect the disease trajectories described by Lunney, Lynn and colleagues [21], irrespective of the underlying life-limiting illness.

### Other measures of performance

KPS is not the only measure of performance status used in palliative care and oncology. The shorter Eastern Cooperative Oncology Group (ECOG) performance status scale was derived from the KPS [22]. It is only occasionally used as a main outcome in clinical trials in the palliative care setting since the 5-item scale inadequately differentiates between patients with poor functional status. Similar concerns about the KPS having limited sensitivity to monitor change when patients score at the low end of the scale have been reported by other authors [2]. In this current study, AKPS was superior to KPS and TKPS in the lower range of the scale and provided more categorical levels of performance status than the ECOG scale.

In 1996, Anderson *et al *described the Palliative Performance Scale (PPS), a modification of the KPS based on 5 observable parameters (ambulation, activity combined with evidence of disease, self-care, intake and conscious level) scored into 11 categories. [23]. PPS predicted time to death (mean 162 days) for a population of Australian patients admitted to a palliative care unit[24]. PPS was not as predictive of survival for a similar group of Japanese palliative care patients with a mean survival of 49 days [11]. AKPS is a less complex measure which is easier to use with each clinical encounter.

Other scales such as the Edmonton Functional Assessment Tool extend on the functional parameters described in the KPS, PPS and TKPS [2], however as these scales and their scoring becomes more complicated, their day-to-day applicability decreases. AKPS focuses on current functional abilities and on changes in function if used longitudinally; it provides an important parameter in the overall assessment of any person with a life-limiting illness.

### Limitations

This study is representative by age and gender for palliative care in Australia. Because it was a sub-study of a larger randomized controlled trial where pain in the previous 3 months was an inclusion criterion, the population almost all had cancer as their life-limiting illness (92%) versus 85% seen in the general population referred to the palliative care service. Given that KPS was originally developed for people with cancer and had been extrapolated to other clinical settings (AIDS, end-stage organ failure), this should not be a major limit to generalizing these findings. In the early parts of the trial, measurement of KPS, TKPS and AKPS was predominantly in the community setting limiting the ability to observe its utility in other care venues. As more trial participants were hospitalized over time, data were collected from the inpatient settings therefore reflecting the range of settings in which palliative care is delivered. Many assessments were in the upper range of the scales; only a minority of patients were bedridden. An evaluation on a palliative care unit with more severely disabled patients might show other results. The missing correlation of KPS and TKPS with survival in the lower range of the scale may have been biased by small patient numbers in these clusters. Also, ideally none of the performance status measures would have any reference to the amount of health services required at any of the levels. AKPS has considerably less reference, but still states "requires... frequent medical care" in its description of AKPS 50.

Inter-rater reliability evaluation of the AKPS was originally planned as part of this sub-study. Ill palliative care patients were overly burdened by multiple visits on the same day for research data collection. An alternative plan was enacted with collection of measures after the informed consent document was signed and then comparing these results with those reported on the baseline assessment within 48 hours of the consent visit. Unfortunately many patients were too unstable or the timing of the baseline assessment was too far from the consent visit; there were not enough data available for these analyses. The inter-rater reliability and other psychometric properties of the KPS has previously been documented[3,6,7]. As AKPS was more predictive of survival outcomes than KPS, the reliability was expected to be better if it differed from that reported for the KPS.

In addition to being more predictive of survival at the lower end of the scale, the AKPS may be more appropriate than the other performance status scales in settings outside of cancer. Ninety-two percent of participants in this study had cancer, so evaluation of the AKPS in non-cancer diagnoses was limited. Further work should concentrate on disabled palliative care patients whose performance status is at the lower end of the scale. Future studies will focus on validity outside of the cancer setting and with more diverse palliative care populations. Also, the performance of the AKPS will be compared to other performance status measurement tools appropriate for palliative care such as the ECOG scale, PPS and Edmonton Functional Assessment Tool.

## Conclusion

The AKPS is an important contemporary modification to the KPS that incorporates language more appropriate to current clinical care without limiting it to judgments on intensity of clinical treatment or resources available. The AKPS assists with clinical decision-making across a range of clinical settings. The ability of staff to relate to the performance status scale used will continue to be a key factor in uptake and use of a measure that has such broad application. It is appropriate to use the AKPS as a primary outcome variable in the Palliative Care Trial.

## Competing interests

The author(s) declare that they have no competing interests.

## Authors' contributions

*APA: *Conception and design, data preparation, data analysis, analysis interpretation, preparation of the first and subsequent manuscript drafts, and final approval of the manuscript

*T S-J: *Conception and design, data collection, data preparation, data analysis, analysis interpretation, review of manuscript drafts, and final approval of the manuscript

*BSF: *Conception and design, analysis interpretation, review of manuscript drafts, and final approval of the manuscript

*DW: *Developed the AKPS, approval of design of the validation study, analysis interpretation, review of manuscript drafts, and final approval of the manuscript

*DCC: *Conception and design, analysis interpretation, preparation of the first and subsequent manuscript drafts, and final approval of the manuscript

## Pre-publication history

The pre-publication history for this paper can be accessed here:


